# Randomized Phase II trial assessing estramustine and vinblastine combination chemotherapy *vs* estramustine alone in patients with progressive hormone-escaped metastatic prostate cancer

**DOI:** 10.1038/sj.bjc.6601468

**Published:** 2004-01-06

**Authors:** W Albrecht, H Van Poppel, S Horenblas, G Mickisch, A Horwich, V Serretta, G Casetta, J M Maréchal, W G Jones, S Kalman, R Sylvester

**Affiliations:** 1 Department of Urology, Rudolfstiftung, Juchgasse 25, Vienna A 1030, Austria; 2 Department of Urology, UZ Gasthuisberg, Leuven, Belgium; 3 Department of Urology, Antonie Van Leeuwenhoek Ziekenhuis, Amsterdam, The Netherlands; 4 Department of Urology, Erasmus Rotterdam, The Netherlands; 5 Department of Oncology, Royal Marsden, Surrey, UK; 6 Department of Urology, Universita di Palermo, Italy; 7 Department of Urology, Ospidale Molinette, Torino, Italy; 8 Department of Urology, Edouard Herriot, Lyon, France; 9 Department of Oncology, Cookridge Hospital, Leeds, UK; 10 EORTC Data Center, Brussels, Belgium

**Keywords:** hormone-escaped prostate cancer, Phase II, EMP/VBL vs EMP

## Abstract

Based on the results of combined data from three North American Phase II studies, a randomised Phase II study in the same patient population was performed, using combination chemotherapy with estramustine phosphate (EMP) and vinblastine (VBL) in hormone refractory prostate cancer patients. In all, 92 patients were randomised into a Phase II study of oral EMP (10 mg kg day continuously) or oral EMP in combination with intravenous VBL (4 mg m^2^ week for 6 weeks, followed by 2 weeks rest). The end points were toxicity and PSA response in both groups, with the option to continue the trial as a Phase III study with time to progression and survival as end points, if sufficient responses were observed. Toxicity was unexpectedly high in both treatment arms and led to treatment withdrawal or refusal in 49% of all patients, predominantly already during the first treatment cycle. The mean treatment duration was 10 and 14 weeks, median time to PSA progression was 27.2 and 30.8 weeks, median survival time was 44 and 50.9 weeks, and PSA response rate was only 24.6 and 28.9% in the EMP/VBL and EMP arms, respectively. There was no correlation between PSA response and survival. While the PSA response in the patients tested was less than half that recorded in the North American studies, the toxicity of EMP monotherapy or in combination with VBL was much higher than expected. Further research on more effective and less toxic treatment strategies for hormone refractory prostate cancer is mandatory.

The median duration of response of metastatic prostate cancer to androgen ablation is approximately 12–16 months (Crawford *et al*, 1989). The management of hormone escaped metastatic prostate cancer is difficult and there is no standard therapy at the present time. The median survival time is only 9–12 months. The evaluation of new agents in metastatic prostate cancer by the Genito-Urinary Group of the European Organisation on Research and Treatment of Cancer (EORTC GU Group) has taken the form of a series of Phase II studies using measurable soft-tissue metastases as indicator lesions. Based on a 29% partial response rate in a previous study of mitomycin C (MMC) (Jones *et al*, 1986), the EORTC GU Group undertook a Phase III trial comparing MMC with estramustine in an unselected group of hormone-escaped metastatic patients. This showed that both agents were much more toxic than suggested by previous Phase II trials, and failed to confirm the previous response rate (Newling *et al*, 1993). Although Phase II studies with measurable lesions are necessary to investigate possible activity in hormone refractory prostate cancer, for practical purposes, testing for activity and toxicity must be undertaken in the more common situation of hormone-escaped disease with bone metastases and local progression rather than just in patients with less common lymph node and other soft tissue metastases.

Estramustine, a combination of an oestrogen with a nor-nitrogen mustard, disrupts the cytoplasmatic microtubules, inhibits the assembly of the nuclear matrix and the multidrug resistance transporter p-glycoprotein (Mareel *et al*, 1988; Perry and McTavish, 1995). Estramustine phosphate (EMP) is commonly used for the treatment of hormone refractory prostate cancer and historical data have shown a clinical response rate of about 30% (Van Poppel and Baert, 1991). In all, 18 Phase II studies of single-agent estramustine in 634 patients showed objective measurable responses in only 19% (Benson and Hartley-Asp, 1990). Vinblastine (VBL) has been shown to induce objective tumour regression in 21% (Dexeus *et al*, 1995). Estramustine phosphate and the vinca-alcaloids inhibit the cellular microtubular apparatus by distinct molecular mechanisms, suggesting that the cytotoxic effect of the combination could exceed that of each single agent. This additive and possible synergistic effect has been observed *in vitro* (Benson and Hartley-Asp, 1990).

Combined data from the Memorial Sloan Kettering Cancer Center (Seidman *et al*, 1992), Fox Chase Cancer Center (Hudes *et al*, 1992) and MD Anderson (Amato *et al*, 1995) showed that seven of 19 patients with measurable hormone refractory prostate cancer responded to combined EMP and VBL chemotherapy. Regression was noted for lymph nodes and lung metastases. Overall partial remission was reported in 42% of 83 patients with measurable disease and a decrease in serum PSA greater than 50% was noted in 46 out of 83 patients treated in similar Phase II studies at the three centres. As shown by these investigators and in a previously published paper by Scher *et al* (1990), a 50% decrease in PSA may be used as a parameter for response evaluation and may also be a surrogate for survival. If a strong correlation between PSA decrease and treatment response could be shown, this would help to identify those patients who will have the most benefit from chemotherapy as well as to identify the worst subgroup, in which an ineffective and possibly toxic treatment could be stopped early.

On the basis of the albeit limited data and the absence of an effective alternative therapy the EORTC-GU Group initiated a randomised Phase II study assessing EMP plus VBL combination chemotherapy *vs* EMP alone, the latter still being a generally accepted treatment in some European centres, to determine the PSA response rate and the toxicity of these treatments in patients with hormone-escaped, progressive, metastatic prostate cancer.

## MATERIALS AND METHODS

### Eligibility

Patients with histologically proven metastatic prostate cancer showing evidence of disease progression based on a rising serum PSA (doubling of the lowest ever evaluated PSA value=nadir) and/or new metastases demonstrated by appropriate imaging techniques, or deterioration of performance status, or pain or weight loss due to prostate cancer despite sustained previous hormone therapy were eligible. The minimum PSA level required was 10 ng ml or ⩾2.5 × normal PSA.

As EMP also lowers the testosterone levels, only patients who had undergone sufficient antiandrogen therapy (orchiectomy, LHRH) with serum testosterone at castrate level prior to progression were included. Patients taking oral antiandrogen monotherapy were ineligible. Patients treated with total androgen blockade had to stop antiandrogens at least 4 weeks prior to trial entry and were eligible only if there was evidence of progression 4 weeks after the cessation of the antiandrogen. LHRH agonist depot injections were maintained.

Prior radiation therapy to the primary and for metastases, as well as the systemic administration of radionuclides, was allowed. Patients of all ages with a life expectancy of more than 90 days and a WHO performance status of 0–2 were included. An adequate bone marrow reserve was required with a white blood cell count ⩾3.0 × 10^9^ l and platelet count ⩾100 × 10^9^ l. If, however, a lower count was judged to be due to tumour invasion of the bone marrow, treatment could be initiated. Adequate renal (creatinine ⩽1.5 × normal) and liver (bilirubin ⩽1.5 × normal) functions were also required.

Eligibility criteria included no prior treatment with systemic cytotoxic drugs, corticosteroids or steroid-containing drugs. Previous second malignancies (except treated basal cell carcinoma of the skin or other cancers inactive for more than 1 year), systemic congestive heart failure, angina pectoris, myocardial infarction or stroke within the previous 6 months, uncontrolled hypertension, deep venous thrombosis and active uncontrolled infection were additional exclusion criteria.

### Trial design

The study was a randomised Phase II trial simultaneously screening two treatment regimens. Patients were randomised to receive either EMP and VBL combination chemotherapy (Group 1) or EMP alone (Group 2). The end points were the overall response rate monitored by PSA response, toxicity and quality of life.

If, after this Phase II study, the toxicity was considered to be acceptable and if the estimated response rate on the combination arm was at least as high as in the monotherapy arm, the trial would be continued as a randomised Phase III study, taking the duration of survival as the main end point.

Central registration, randomisation, and data collection were undertaken at the EORTC Data Center in Brussels. All participating centres had to provide evidence of local ethics committee approval before being allowed to enter patients. Patient-informed consent was required before randomisation. Stratification at randomisation was performed for institution and performance status.

### Investigations

Pretreatment investigations included standard haematologic and biochemical parameters, serum PSA level and performance status, and these were to be repeated after every cycle; the site and estimated number of metastases, chest X-ray and bone scan were to be repeated after every two cycles. Quality of life was assessed by the EORTC QLQ-C30(+3) questionnaire plus the prostate cancer module before the start of the treatment, and repeated after every two cycles of 8 weeks each.

### Chemotherapy

Estramustine phosphate was given daily and administered orally at a calculated dose of 10 mg kg^−1^ day. As each capsule contained 140 mg EMP, the calculated dose was divided by 140. This quotient was rounded to the nearest integer to give the total number of capsules, which were to be taken divided into three daily doses at 1000, 1500 and 2200 h at least 2 h before or after food, milk, milk products, antacids or calcium-containing substances, which would impair the absorption of EMP. Vinblastine 4 mg m^−2^ (to a maximum dose of 7 mg) was given by i.v. push injection weekly for 6 weeks, followed by 2 weeks rest. The cycles were repeated every 8 weeks. Concomitant antiemetics (5HT3-antagonists) were routinely administered.

Dose modification of VBL in the case of hamatologic toxicity and neurotoxicity followed conventional guidelines. Reduction of the dose of EMP was permitted for gastrointestinal toxicity, but was not recommended for paresthesias, diarrhoea or hepatic enzyme elevation. For both drugs, the following dose modifications were recommended: In the case of grade 2 toxicity, a reduction to 75%, in the case of grade 3 or 4, treatment was withheld. If toxicity was resolved by the next treatment, VBL dose was determined by the worst grade of toxicity experienced. If Grade 2, treatment was continued at 75%, if Grade 3 or 4, treatment was continued at 50% of the last dose received.

In the case of a PSA increase, the treatment was continued until PSA progression was confirmed and, if possible, until the symptoms dictated a change of therapy. The likelihood of response to third-line therapy was considered small, and therefore an asymptomatic increase of PSA was not a reason for treatment withdrawal.

Patients with an objective response or no change with a stable performance status were maintained on treatment until objective evidence of disease progression based on PSA or significant toxicity developed. If a complete response was achieved, treatment was continued, if toxicity allowed, for at least three more courses.

### Response evaluation

Except for patients who were progressive after one course, patients were evaluable for response only if they had been treated for a minimum of two 8-week cycles of therapy. Patients were evaluable for toxicity if they had started treatment. Toxicity was documented using the Common Toxicity Criteria (CTC-NCIC). Evidence of response was documented exclusively on the basis of PSA levels: Complete response: Normalisation of PSA (less than 4.0 ng ml, Hybritech). Partial response: decrease from baseline PSA value by 50% or more, but without normalisation. Progression: increase by 50% or more from nadir PSA. Stable: all patients who did not meet response or progression. The overall response was the best response at or after 8 weeks.

### Statistical methods

The minimum PSA response rate of interest was 40%. 20 patients were to be initially treated in each arm. Thereafter, five additional patients were to be treated in each arm for each PSA response seen in the first 20 patients in each arm, up to a maximum of 40 patients in each arm. Time to PSA progression and duration of survival were estimated using Kaplan–Meier technique. As this was a Phase II trial, no formal comparisons were made and no *P*-values are provided.

## RESULTS

In all, 92 patients, 46 on each treatment arm, were entered between October 1995 and November 1996 by 28 institutions, half of them by eight centers.

### Patients characteristics

All together, 90 patients were eligible. Two were ineligible, one in each treatment group: one patient had no metastases and the other had a PSA level <10 ng ml. The two groups were balanced with respect to age, pain at entry and known prognostic factors, except that patients on the combination of EMP and VBL had slightly more bone metastases at entry (Table 1

**Table 1:** General patient characteristics (all eligible patients)

	**EMP & VBL**	**EMP alone**
***n*=**	**45**	**45**
New mets within the last 4 weeks	38 (82.4%)	35 (77.8%)
Deterioration of performance status, pain or weight loss	36 (80.0%)	37 (82.2%)
		
*WHO performance status*		
0	7 (15.6%)	8 (17.8%)
1	28 (62.2%)	29 (64.4%)
2	10 (22.2%)	8 (17.8%)
		
*Degree of pain from metastases*		
None	7 (15.6%)	7 (15.6%)
Mild	15 (33.3%)	11 (24.4%)
Moderate	11 (24.4%)	15 (33.3%)
Severe	11 (24.4%)	11 (24.4%)
Intractable	1 (2.2%)	—
Unspecified	—	1 (2.2%)
		
Recent weight loss >5 kg	8 (17.8%)	6 (13.3%)
*Bone mets at entry*		
None	4 (8.9%)	3 (6.7%)
<5	4 (8.9%)	11 (24.4%)
5–15	19 (42.2%)	13 (28.9%)
>15 superscan	18 (40.0%)	18 (40.4%)
		
Visceral mets	12 (26.7%)	13 (28.9%)
		
*Pretreatment PSA*		
2.5–10 × normal	10 (22.2%)	13 (28.9%)
10–25 × normal	11 (24.4%)	7 (15.6%)
25–100 × normal	9 (20.0%)	14 (31.1%)
100–1000 × normal	15 (33.3%)	11 (24.4%)
		
*Type of previous hormonal treatment*		
LHRH agonists	28 (62.2%)	25 (55.6%)
Orchiectomy	18 (40.0%)	22 (48.9%)
Oestrogens	4 (8.9%)	—
MAB	12 (26.7%)	12 (26.7%)
Antiandrogens	27 (60.0%)	21 (46.7%)
Other	3 (6.7%)	2 (4.4%)
		
*Time since the first histological confirmation of prostate cancer*		
Minimum	0.5 years	0.4 years
Median	2.6 years	2.7 years
Maximum	10.7 years	13.2 years

).

### Treatment duration

Two patients on the combination arm received no treatment. Only 31.1% of the EMP/VBL patients and 42.2% of the patients on EMP alone received two or more 8 weeks cycles. An additional 22.2 and 15.6% got between one and two cycles, respectively. The main reasons for stopping were toxicity, treatment refusal by the patients, progression according to the protocol or clinical progression (Table 2

**Table 2:** Reasons for stopping treatment (eligible patients who started treatment)

	**EMP & VBL**	**EMP alone**
***n*=**	**43**	**45**
PSA progression	11 (25.6%)	13 (28.9%)
Death due to malignant disease	—	1 (2.2%)
Toxicity	13 (30.2%)	14 (31.1%)
Treatment refusal	9 (20.9%)	8 (17.8%)
Intercurrent death	1 (2.3%)	—
Clinical progression	9 (20.9%)	9 (20.0%)

). The median treatment duration was 10 and 14.3 weeks, respectively (Figure 1

**Figure 1:**
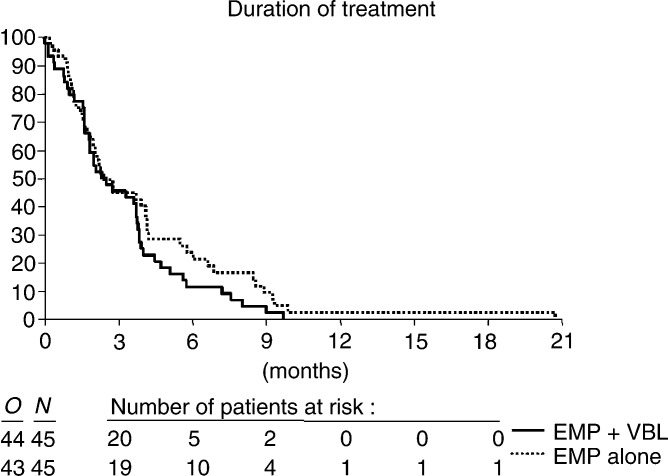
Duration of treatment.

).

### Toxicity

Among all eligible patients who received treatment, grade 1 and 2 myelosuppression occurred in 11.6%. on the combination arm and in 4.5% of the EMP alone arm. In total, 27.9 and 26.7%, respectively, suffered from cardiovascular toxicity. Two deaths possibly related to treatment occurred in the combination arm (one myocardial infarction and one cerebrovascular accident). In the two groups, nausea was reported in 44.2 and 55.6%, vomiting in 18.6 and 35.6%. Neurological toxicity was seen in 27.9 and 28.9%, liver function impairment was noticed in 9.3 and 22.2% (bilirubin) and 32.6 and 24.4% (SGOT), respectively. A total of 11 patients in the combination arm had dose reductions (six due to haematological toxicity, three gastrointestinal, two neurological) and six patients in the EMP arm for gastrointestinal toxicity. Details are given in Table 3a and b

**Table 3:** Toxicities of EMP/VBL and EMP (NCIC-CTG)

	**No of patients with NCIC-CTG grade toxicity**				
**Type of toxicity**	**1**	**2**	**3**	**4**	**%**
*(a) EMP/VBL*					
Cardiovascular^a^	1	5	4	2^b^	27.9
Gynecomastia	13	1			32.6
Diarrhoea	4	1	2		16.3
Nausea	8	8	3		44.2
Vomiting	5	3			18.6
Other gastrointestina^c^	3	3	1		16.3
Alopecia	4				9.3
Neurological^d^	6	4	2		27.9
Bilirubin rise		2	1	1	9.3
SGOT rise	13	1			32.6
Platelets	8	1			20.9
Haemoglobin	18	15	1		76.7
WBC	4	1			11.6

*(b) EMP*					
Cardiovascular^e^	3	5	4		26.7
Gynecomastia	6	7			28.9
Diarrhoea	6	1	1		17.8
Nausea	9	11	5		55.6
Vomiting	9	5	1	1	35.6
Other gastrointestinal^f^	4	3	1		17.8
Alopecia	4	1			11.1
Neurological^g^	3	7	3		28.9
Bilirubin rise		5	3	2	22.2
SGOT rise	9	2			24.4
Platelets	6	1	1		17.8
Haemoglobin	23	8	2		73.3
WBC	2				2.2

a Cardiovascular toxicity: venous thrombosis 2, oedema, pulmonary embolism, myocardial infarction, atrial fibrillation, insufficient cardiac function.

b 2 deaths (myocardial infarction, stroke).

c Two other gastrointestinal toxicity: abdominal pain 2, sensitive oesophagus, pyrosis.

d Neurological toxicity: constipation 4, depression 2, muscle cramping 2, paresthesia.

e Cardiovascular toxicity: oedema 7, venous thrombosis 2, pulmonary embolism, atrial fibrillation, insufficient cardiac function.

f Other gastrointestinal toxicity: anorexia 4, abdominal pain 3.

g Neurological toxicity: constipation 8, depression, muscle cramping, paresthesia.

.

### Response to treatment

The PSA response data are summarized in Table 4

**Table 4:** PSA response to treatment (all eligible patients)

	**EMP & VBL**	**EMP alone**
***n*=**	**45**	**45**
CR	1 (2.2%)	3 (6.7%)
PR	11 (24.4%)	10 (22.2%)
SD	20 (44.4%)	18 (40.0%)
Progression	4 (8.9%)	7 (15.6%)
Early death	3 (6.7%)	1 (2.2%)
Not assessed	6 (13.3%)	6 (13.3%)

**Figure 2:**
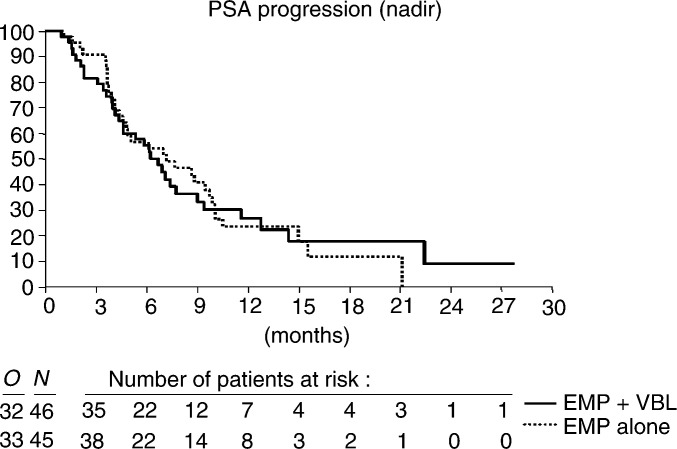
Time to PSA-progression from nadir.

**Figure 3:**
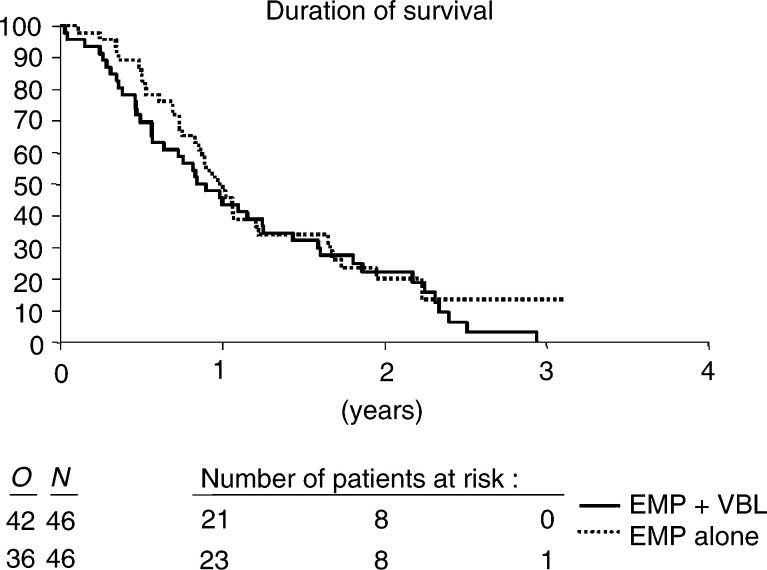
Duration of survival.

**Figure 4:**
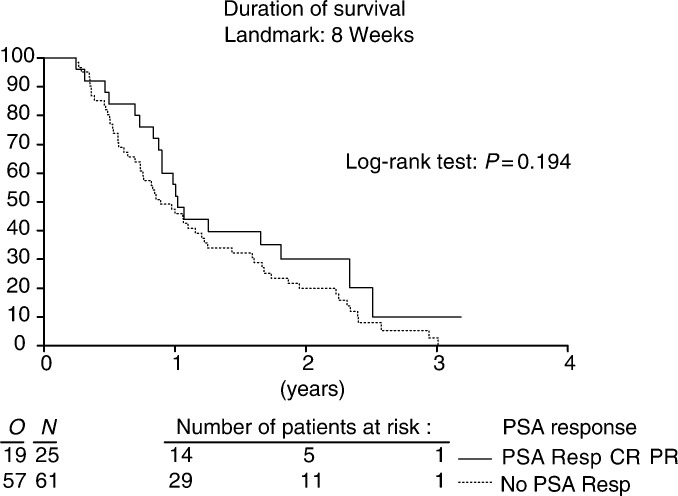
Duration of survival in respect to PSA response.

. The PSA-response rate in all eligible patients was 24.6 and 28.9% in the two arms. The median time to PSA progression from the nadir was 27.2 and 30.8 weeks (Figure 2

). In all, 78 patients had died at the time of the final analysis, median survival was 44 and 50.9 weeks, respectively (Figure 3

). No correlation could be found between the extent of PSA response and survival. The median survival of the PSA responders was 12.2 months as compared to 10.7 months for the nonresponders (*P*=0.194) (Figure 4

).

## DISCUSSION

Traditionally, the assessment of toxicity and activity of drugs used in a new indication has to be performed in patients with measurable disease. Prostate cancer, mainly metastases to bone and skeletal metastases, are not considered as measurable lesions. Measurable metastatic prostate cancer occurs in only about 10% of patients and the results obtained in this specific group might not compare with those obtained in patients with bone metastases. Patients with lymph node and soft tissue metastases respond much better to therapeutic interventions and seem to tolerate chemotherapy better than those with bone metastases. This has led several groups and a consensus conference to accept PSA as a parameter for measuring treatment activity (Scher *et al*, 1990; Kelly *et al*, 1993; Dawson and McLeod, 1997; Smith *et al*, 1998; Bubley *et al*, 1999).

The strongest evidence to support a role for PSA as a marker of response and as a surrogate end point is the correlation between the magnitude of PSA decline and survival. Kelly *et al* (1993) demonstrated a significant improvement in median survival in patients with a 50% or greater decline as compared with a decline of less than 50% in PSA after treatment. These analyses were inherently biased because they were subgroup comparisons among patients with probably different prognoses. Wide fluctuations were seen in PSA values by Scher *et al* (1990), indicating a transient effect of the drugs on PSA production. Thus, several authors expressed reservations to use PSA as a surrogate end point for survival (Bauer *et al*, 1999; Sridhara *et al*, 1995; Verbel *et al*, 2002). Nevertheless, most current studies in metastatic prostate cancer patients include PSA responses as primary or secondary end points (Dawson and McLeod (1997); Smith *et al*, 1998). This approach was also used in the previously mentioned studies (Hudes *et al*, 1992; Seidman *et al*, 1992; Amato *et al*, 1995) as well as in this trial. As mentioned in the Results section, in our trial there was no correlation between PSA response and survival.

EMP has been used in Europe as the first choice for the treatment of hormone-escaped metastatic prostate cancer (Van Poppel and Baert, 1991). A previous EORTC Phase III study has emphasised its limited activity and the relevant toxicity (Newling *et al*, 1993). The three North American reports on the combination of EMP and VBL suggested better efficacy, and good compliance with acceptable toxicity (Hudes *et al*, 1992; Amato *et al*, 1995; Seidman *et al*, 1992). A phase III study by Hudes *et al* (1999) comparing vinblastine to vinblastine plus estramustine, using a similar design as ours, reported a significant advantage with the combination for progression-free but not overall survival in 193 patients. While gastrointestinal toxicity was worse with the adition of estramustine, interestingly, there was significantly less granulocytopenia in the VBL plus EMP arm.

Therefore, the EORTC GU-Group started a randomised Phase II trial with the option of extending the study to a Phase III trial if these results were confirmed.

However, our trial was not able to reproduce these results, although the protocol was designed in the same way. The main problem was the fact that only 31.1% of patients in the combined agent arm and 42.2% of the patients in the EMP alone arm were able to receive two or more cycles. The reason for early stopping was toxicity (30.2 and 31.1%) or refusal by the patients (20.9 and 17.8%). Progression led to early cessation of treatment in 44.2 and 46.7%. This is in contradiction to the cited North American studies, although their toxicity was equal to ours. Whether there are differences in overall tolerance between the two continents or if the European patients were treated at a later stage and therefore were less well remains unclear. Moreover, about 20% of all patients were taken off study because of clinical progression not according to the protocol, for example, not meeting the criteria of PSA progression. There was no difference in the stopping policy whether the treating physician was a urologist or a medical oncologist. We were also unable to reproduce the encouraging results in PSA decrease as quoted in the North-American trials, as our patients had less than half of the reported PSA response rate.

Recent analyses of EMP toxicity and pharmacology (Perry and McTavish, 1995) have suggested that this drug may better be administered over a limited number of days around the time of the other cyclical anticancer drugs, rather than on a continuous daily schedule, and this is supported by clinical studies (Petrylak *et al*, 1999; Savarese *et al*, 1999; Sinibaldi *et al*, 1999).

In this trial, both regimens with continuous daily EMP doses caused a severe deterioration of the patients' well being. Since the patients are in need of palliation, the main goal of the treatment should be to improve the quality of life. This goal could not be achieved with either tested regimen. Based on these results, continuation of the study as a randomised phase III trial was not justified.

## CONCLUSION

The PSA response rate in the patients treated with either regimen was low, less than 30%, and toxicity was clinically relevant. Daily continuous EMP monotherapy or in combination with VBL, therefore, cannot be recommended as standard second-line treatment when metastatic prostate cancer has become hormone refractory. Further research on more effective and less toxic regimens is needed.
